# Antagonism of Innate Immunity by Paramyxovirus Accessory Proteins

**DOI:** 10.3390/v1030574

**Published:** 2009-10-28

**Authors:** Raychel Chambers, Toru Takimoto

**Affiliations:** Department of Microbiology and Immunology, University of Rochester Medical Center, Rochester, NY 14642, USA; E-Mail: raychel_chambers@urmc.rochester.edu

**Keywords:** interferon, paramyxoviruses, innate immunity, apoptosis

## Abstract

*Paramyxovirinae*, a subfamily of *Paramyxoviridae*, are negative strand RNA viruses comprised of many important human and animal pathogens, which share a high degree of genetic and structural homology. The accessory proteins expressed from the P/V/C gene are major factors in the pathogenicity of the viruses, because of their ability to abrogate various facets of type I interferon (IFN) induction and signaling. Most of the paramyxoviruses exhibit a commonality in their ability to antagonize innate immunity by blocking IFN induction and the Jak/STAT pathway. However, the manner in which the accessory proteins inhibit the pathway differs among viruses. Similarly, there are variations in the capability of the viruses to counteract intracellular detectors (RNA helicases, mda-5 and RIG-I). Furthermore, a functional specificity in the antagonism of the IFN response has been reported, suggesting that specificity in the circumvention of innate immunity restricts viral host range. Available evidence indicates that paramyxoviruses employ specific strategies to antagonize the IFN response of their specific hosts, which is one of the major factors that determine viral pathogenicity and host range.

## 1. Introduction

The interferon (IFN) system is the first line of defense against viral infection in mammals. This system is designed to block the spread of virus infection in the body. Detection of virus infection by intracellular receptors (sensors) results in activation of multiple pathways, leading to the production of IFN-α/β, which activates the Jak/STAT signaling pathway and stimulates transcription of a variety of antiviral genes in neighboring cells. Many of the pathogenic viruses, however, have evolved mechanisms to evade the IFN system by blocking IFN synthesis, the signaling pathway or both. In the case of paramyxoviruses, accessory proteins encoded by the P/V/C gene mediate antagonism of innate immunity. Mutant viruses unable to express the accessory proteins are attenuated *in vivo*, indicating the critical role of the accessory proteins in viral growth and pathogenicity. Although most of the paramyxoviruses are able to antagonize IFN activity, the mechanism and proteins responsible for the antagonism differs among the viruses. This minireview describes a variety of anti-innate activities mediated by accessory proteins of viruses belonging to the *Paramyxoviridae* subfamily, with the exception of henipavirus, which will be covered in a different section of this issue.

## 2. General features of paramyxoviruses

Family P*aramyxoviridae,* in the order *Mononegavirales,* is composed of a wide range of “classic” and “emerging” viruses of medical or veterinary significance. The family is categorized into two subfamilies, *Paramyxovirinae* and *Pneumovirinae*, according to several criteria, such as morphology, genomic organization, role of the encoded viral proteins and the sequence relationship between these proteins. Subfamily *Paramxyxovirinae*, which is the focus of this review, consists of five genera: *Respirovirus*, *Morbillivirus, Rubulavirus*, *Avulavirus* and *Henipavirus*. The well-studied viruses belonging to this subfamily include Sendai virus (SeV) and human parainfluenza virus types 1 and 3 (hPIV1 and 3)(*Respirovirus*), parainfluenza virus 5 (PIV5, formerly SV5) and mumps virus (MuV)(*Rubulavirus*), measles virus (MeV)(*Morbillivirus*), Newcastle disease virus (NDV)(*Avulavirus*) and Hendra virus (HeV) and Nipah virus (NiV) (*Henipavirus*).

Paramyxoviruses contain nonsegmented negative-strand RNA genomes. Their genomes are 15–19 kB in length, and the genomes contain six to ten tandemly linked genes. Each gene is flanked by conserved transcriptional control sequences, which are linked by an intergenic sequence. The viral mRNAs are transcribed monocistronically, resulting in one protein being expressed from a single mRNA, except the P gene, as described below. Viral RNA is encapsidated with nucleocapsid (N) protein, which is associated with a polymerase complex composed of phospho- (P) and large (L) proteins. The virion contains a lipid bilayer envelope that is derived from the plasma membrane of the host cell. Inserted into the viral envelope are two surface glycoproteins responsible for virus attachment and membrane fusion. Residing between the envelope and the core is the viral matrix protein that is important in virion assembly and incorporation of nucleocapsid into progeny virions. Paramyxovirus replication takes place entirely in the cytoplasm, and progeny virions are formed at the plasma membrane of infected cells [1].

## 3. Paramyxovirus accessory proteins expressed from the P gene

**Figure 1.**
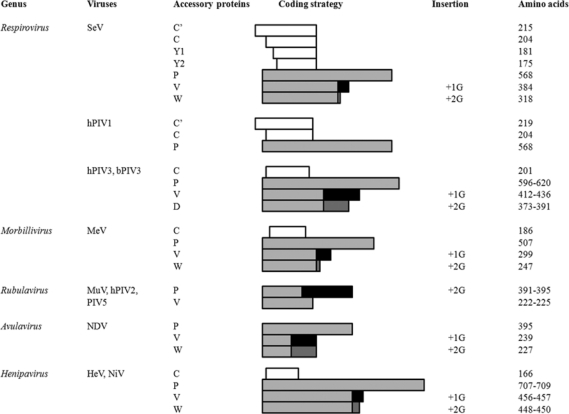
Accessory proteins expressed from *Paramyxovirinae*. The V, W, and D mRNAs are generated by RNA editing, in which one or two G residues are inserted into the transcripts of the P gene at the editing site, except *Rubulaviruses*, which produce V from intact mRNA and P from edited mRNA. In addition to V/W/D proteins, *Respirovirus*, *Morbillivirus* and *Henipavirus* express C proteins from ORFs overlapping the amino-terminal portion of the P ORF in the +1 frame.

Most mRNAs of *Paramyxovirinae* are translated into one protein, with the exception of transcripts from the P gene, which express accessory proteins, in addition to the P protein. Non-rubulavirus *Paramyxovirinae* P proteins are expressed from a faithful transcript of the P gene, and all the viruses in this group express at least one accessory protein (Figure 1). They are expressed by two different strategies: one by RNA editing for V/W/D expression, and the other by overlapping open reading frame (ORF) for C expression. All the viruses of *Paramyxovirinae* encode a characteristic editing site in the P gene except hPIV1, and the number of inserted G residues, as well as the frequency of G nucleotide insertion is determined by sequences surrounding and within the editing site [2–4]. All the V proteins are expressed from edited RNA containing a single G residue inserted at the editing site, except *Rubulavirus* that produces V protein from intact unedited mRNA, and edited RNA with 2 inserted G residues translate the P protein. The V protein is a ∼25- to ∼30-kDa polypeptide that shares an N-terminal domain with the P protein but has a distinct C-terminal domain as a result of frame shift due to inserted G nucleotide(s). The C-terminal V-specific domain is highly conserved among related paramyxoviruses, with spaced histidine and cysteine residues forming a zinc-binding domain, and indeed binds two atoms of zinc molecules per V protein [5–7]. *Respiroviruses, Morbilliviruses, Avulaviruses* and *Henipaviruses* express W/D proteins from mRNAs with two inserted G residues. The relative abundance of these proteins is low compared to the total P mRNA [8]. The function of the W and D proteins is not clear, however, disruption of both V and D proteins of hPIV3 was shown to affect *in vivo* hPIV3 replication [9], while disruption of the W protein had little effect on NDV growth in cultured cells [10].

In addition to RNA editing, *Respiro*-, *Morbilli*- and *Henipaviruses* use alternative translation initiation codons to yield the C proteins (Figure 1). The number of expressed C proteins differs among paramyxoviruses, ranging from 1 to 4. SeV expresses a nested set of C proteins, C’, C, Y1 and Y2, that range in size from 175 to 215 residues. The ORFs are located upstream of the editing site and overlap the P ORF in the +1 frame. The initiation codons of C’, C, Y1, and Y2 are ACG^81^, AUG^114^, AUG^183^ and AUG^201^, respectively. Translation of each of the C’, C, Y1, and Y2 ORFs is terminated at the same downstream stop codon; therefore, these proteins share a common C-terminal region. The hPIV1, which is highly homologous with SeV expresses only C’ and C. The hPIV3 and bPIV3 encode a single species of C protein composed of 199 and 201 residues, respectively. MeV also expresses a single species of C protein with 186 amino acids [11]. HeV and NiV express C proteins composed of 166 residues, and share 83% identity between the viruses [12].

## 4. Functions of the accessory proteins expressed from the P/V/C gene

C proteins are small basic polypeptides that contribute multiple functions to viral growth [1]. The most prominent roles of C proteins are inhibition of intracellular Jak/STAT IFN signaling pathway and downregulation of viral RNA synthesis [13,14]. Details of the anti-innate activities are described below. Earlier studies showed that SeV C protein binds L protein and inhibits viral mRNA synthesis [15–17]. Also, SeV C proteins have been shown to make significant contributions to viral budding from the plasma membrane [18], possibly by escorting ESCRT to the site of virion formation [19], which is required for pinching off from budding plasma membrane patches. Recombinant SeV that does not express C proteins can be recovered, indicating that C proteins are categorically nonessential gene products [20]. However, replication of this knockout virus was greatly impaired even in tissue culture cells, possibly due to the role of C protein in viral transcription, genome replication, and assembly. As expected, because of the functions of C protein in viral replication and anti-IFN activities, growth of recombinant SeV, hPIV1 and hPIV3 with disrupted C protein expression are severely attenuated *in vivo* [9,21,22]. In the case of MeV, C-deficient virus still grows well in certain culture cells but is less virulent *in vivo* [23]. Changes in pathogenesis of C mutant viruses are likely to be related to the ability of C proteins to inhibit IFN responses, which is discussed in further detail below.

V protein also plays a number of important roles in the virus replication cycle, as evidenced by many studies using recombinant viruses that have been engineered to disrupt expression of the whole V protein or its conserved cys-rich domain. In many cases, these mutant viruses grow well in tissue culture cell lines [24–26], but are severely attenuated for growth *in vivo* or cleared rapidly from infected animals [9,23,25,27], likely due to its capacity as an IFN antagonist.

## 5. Innate immunity

The innate immune response is a non-adaptive host defense that forms early barriers to infectious disease, and acts within minutes of infection to eradicate a pathogen or avoid the spread of infection until the threat can be eliminated by the adaptive immune response [28]. Once the immune system has sensed viral infection, multiple signal transduction cascades are initiated resulting in expression of innate cytokines, including IFNs [29]. IFNs are categorized as type I (IFN-α/β), type II (IFN-γ) or type III (IFN-λ) based on sequence homology and the receptor complex used for signaling. Type I IFNs are secreted in direct response to virus infection and consist of the products of the IFN-α multigene family, predominantly synthesized by hematopoietic cells and the product of the single IFN-β gene, which is synthesized mainly by fibroblasts. The released IFNs bind to cell surface receptors and upregulate the expression of more than 300 cellular proteins. The expression of these proteins confers an antiviral state on the cells, providing the cells with an early defense against viral infections [30].

### 5.1. Intracellular detection of viral infection by RIG-I/mda-5 and induction of IFN

RNA helicases can recognize certain viral infection in cells by detecting viral nucleic acids generated in the cytoplasm of an infected cell. The two RNA helicases, retinoic acid inducible gene I (RIG-I) and melanoma differentiation-associated gene-5 (mda-5) have been shown to be important in detecting infection and inducing IFNs. These proteins contain a carboxyl-terminal DExD/H box RNA helicase domain, which recognizes dsRNA, and two amino-terminal caspase-recruiting domain (CARD)-like regions, which are responsible for recruiting downstream signaling molecules. The inhibition of RIG-I expression by siRNA limits IFN-β induction after poly (I:C) treatment [30,31]. The mda-5 shows similar properties to RIG-I, in that in response to their ligands, RIG-I and mda-5 initiate a signaling cascade that results in the activation of transcription factors to promote the induction of IFN-α/β [32,33]. It is thought that binding of viral RNA stimulates ATPase activity and triggers a major conformational change allowing dimerization, which reveals the CARD domains making the domains available to interact with the downstream adaptor protein Cardif/VISA/MAVS/IPS-1 [34–39]. This leads to activation of both IRF-3 and NF- κB which are required for transcriptional induction of the IFN-β promoter. Upon translocation to the nucleus, these transcription factors bind to the IFN-β promoter cooperatively with the c-jun/ATF-2 transcription factor to form the enhanceosome, which is required for optimal transcription of the IFN-β gene. IFN-β production positively feeds back on the cell to upregulate the IRF-7 transcription factor and enhances further production of IFN.

Extensive studies have been performed to determine whether RIG-I and mda-5 act as parallel sensors or have the ability to sense distinct virus-derived signals. It is becoming clear that viruses generate a variety of different pathogen associated molecular patterns (PAMPs), and mda-5 and RIG-I are differentially sensitive to activation by different viruses. Both mda-5 and RIG-I can be activated by synthetic double-strand RNA (dsRNA) poly (I:C). Studies using knockout mice showed that mda-5 was much more important in regulating the response to poly (I:C) treatment than RIG-I [40]. However, a recent study showed that length of the dsRNA influences whether IFN induction is dependent on mda-5 or RIG-I, with mda-5 being more important for induction by long dsRNA and RIG-I being more important for induction by short dsRNA [41]. RIG-I and mda-5 also differ in their ability to recognize various types of RNA, suggesting that the two helicases recognize different structures within the RNA [34]. Single-stranded RNA (ssRNA) and dsRNA molecules bearing a 5′ triphosphate induce IFN via RIG-I and not mda-5 [42,43]. Since most cellular RNAs are either capped or have a 5′ monophosphate, RNA with 5′ triphosphate is recognized as non-self. The ability to recognize different structures within ssRNA or dsRNA by the helicases explains the importance of mda-5 and RIG-I for IFN induction in response to various RNA virus infections.

### 5.2. IFN signaling: Jak/STAT Pathway

Binding of IFN to its cellular receptor triggers a signaling pathway and induces antiviral proteins. A vast array of cell types express cell surface receptors for type I IFNs. The type I IFN receptors are multimeric transmembrane glycoproteins whose extracellular domains bind IFN and whose cytoplasmic domains bind Jak, STAT and other signaling proteins [44]. The receptor has two major subunits, IFNAR1, a 530 amino acid protein, and IFNAR2c, a 315 amino acid protein [45]. Before activation, the IFNAR1 cytoplasmic tail is bound to tyrosine kinase 2 (Tyk2) and the IFNAR2 subunit associates with tyrosine kinase Jak1, STAT2 and is weakly associated with STAT1 [46–48]. Ligand binding dimerizes the receptor inducing a conformational change, resulting in the phosphorylation of tyrosine 466 on IFNAR1 by Tyk2. Phosphorylation of this tyrosine residue provides a strong docking site for STAT2. Tyk2 is then able to phosphorylate tyrosine 690 on STAT2, with phosphorylation of STAT1 performed by Jak1 on tyrosine 701. Phosphorylated STAT1 and STAT2 form a stable heterodimer, which creates a novel nuclear localization signal [49]. In this way, the heterodimers are translocated into the nucleus and are retained until they are dephosphorylated [50]. Once nuclear translocation of STAT1/STAT2 occurs, the complex binds to IRF-9 (p48) to produce the IFN stimulated gene factor 3 (ISGF3) heterotrimer. This trimeric complex then binds to the IFN-stimulated response element (ISRE), which acts as an enhancer on the 5′ regulatory regions of many of the IFN-responsive gene promoters.

## 6. Antagonism of innate immunity by the accessory proteins

Like most other viruses, paramyxoviruses have evolved mechanisms that allow them to at least partially overcome the IFN response in order to establish a productive infection. They achieve this by reducing the production of IFN from infected cells, blocking IFN signaling or both. Interestingly, accessory proteins that are responsible for anti-IFN activities vary among the viruses, as well as their strategies to achieve these tasks (Tables 1 and 2).

### 6.1. Prevention of IFN production

As discussed above, RNA helicases RIG-I and mda-5 detect viral RNAs and initiate a signaling cascade that results in the activation of transcription factors that promote IFN induction. Recent studies have unveiled the specific mechanism of how paramyxovirus accessory proteins inhibit IFN production. One of the best characterized accessory proteins for this function is PIV5 V protein. PIV5 V blocks activation of the IFN-β promoter by transfected dsRNA, or following infection with a recombinant PIV5 encoding a mutant V protein lacking the cys-rich region [51]. The V protein directly binds mda-5 [52] through the cys-rich region [32], thereby inhibiting the activity of mda-5 [34,52]. A recent study showed that upon dsRNA stimulation, mda-5 and RIG-I form homo-oligomers through their helicase domains. Direct interaction of V protein with mda-5 prevents dsRNA binding and consequent self-association of mda-5 [34]. This ability to bind mda-5 through a conserved cys-rich region of V protein is shared among many paramyxoviruses. V proteins of many different paramyxoviruses, including PIV5, hPIV2, SeV, bPIV3, MuV, MeV and NDV, were examined and all of the V proteins were shown to interact with mda-5 and inhibit IFN induction [32,52,53]. Analysis of the interaction interface revealed the mda-5 helicase C domain as necessary and sufficient for association with V proteins from PIV5, hPIV2, MeV and MuV [54]. These V proteins, however, target mda-5, but not RIG-I. None of the V proteins inhibit IFN induction by RIG-I, except SeV, which showed a moderate inhibitory activity [52]. It is not clear why V protein targets only mda-5, but not RIG-I. It is possible that this specificity is due to the specific pathogen-associated molecular patterns (PAMP) produced in infected cells. It might also be that the viruses, which only produce V protein and not C, may not generate RNAs that are detected by RIG-I, and therefore, it is not necessary to block RIG-I. Another possibility is that RIG-I may not generally be present in the types of cells that these viruses infect. Further studies will be necessary to clarify the PAMPs produced in paramyxovirus-infected cells.

In the case of the *Respirovirus* SeV, inhibition of IFN synthesis by V protein does not appear to be profound *in vivo,* since the level of IFN in the infected mouse lung is not significantly different between infections with V(-) and wild-type (wt) SeV during the early period when their viral loads are similar [55]. Instead, RIG-I has been suggested to play a major role in detection of SeV, bPIV3 and NDV infections [31,33,40,53,56–58]. Infection with NDV or mutant SeV deficient in IFN antagonism because of mutations, induce IFN-β in wt or mda5^−/−^ mouse embryonic fibroblasts (MEFs), but not in RIG-I^−/−^ MEFs [40], suggesting that RIG-I is required for detection of NDV or SeV infection. The activation of RIG-I by SeV infection, as well as 5′-triphosphate RNA, is accompanied by the formation of RIG-I dimers [39,59]. In addition to V protein, SeV and bPIV3 C proteins have been shown to modestly inhibit IFN-β production irrespective of the species of signaling molecules used as an inducer [53,56]. Independent expression of C or V inhibits the dsRNA- or NDV-induced activation of IRF-3 and NF-κB, as well as the IFN-β promoter [60,61]. A study of SeV infection in MEFs showed that it is the C, but not V protein that is primarily responsible for antagonizing IFN induction, and that C acts by countering RIG-I, but not mda-5 [56].

Unlike other paramyxoviruses, hPIV1 does not express V protein. However, hPIV1 C protein has been found to inhibit the activation of IRF-3 and the production of IFN-α/β in monkey and human cells [62]. A mutant virus containing a single mutation at C protein F170S, which does not affect the P protein sequence, strongly activates IRF-3 and induces IFN-β production, indicating hPIV1 C can circumvent IFN production stimulated by hPIV1 infection [62]. It is not known yet, however, whether hPIV1 C targets RIG-I or mda-5. *Morbillivirus* MeV also expresses C protein, however, unlike its V protein, C protein possesses no activity to block the IFN production pathway *per se* [63]. However, recombinant MeV lacking C protein expression strongly stimulates IFN production in spite of the production of V protein, possibly due to aberrant RNA synthesis in infected cells. Therefore, both V and C proteins, directly or indirectly, contribute to the suppression of IFN induction induced by MeV infection [63,64].

### 6.2. Various strategies to block the Jak/STAT pathway

In addition to blocking IFN induction, paramyxovirus V and C proteins are known to block IFN signal transduction by targeting STAT proteins. STATs are essential components in the IFN signaling pathway that induce antiviral proteins in response to IFNs. While it is phenotypically similar in targeting and inhibiting STAT protein functions, the strategies of STAT-directed suppression by accessory proteins are diverse, as discussed below.

One of the approaches used to inhibit IFN signaling by paramyxovirus accessory proteins is proteolytic degradation of STAT proteins. *Rubulaviruses* PIV5, MuV, hPIV2 and *Avulavirus* NDV are known to induce STAT degradation [10,65–67]. It was first reported in a study of PIV5 that STAT1 protein accumulation was dramatically reduced in cells infected with PIV5 or expressing PIV5 V [65]. Chemical proteasome inhibitors prevent V-induced STAT degradation [65,68]. The expression of the *Rubulavirus* V proteins was later demonstrated to induce polyubiquitylation of their target STATs [66,69–72]. These viruses’ V proteins can assemble a ubiquitin ligase complex from cellular components, leading to the destruction of STAT proteins [73]. Interestingly, the specificity of STAT protein targeting differs among the viruses; PIV5 and NDV V target STAT1 for polyubiquitylation and proteasomal degradation [10,65], MuV V protein eliminates both STAT1 [71] and STAT3 [71,74], leaving STAT2 intact, and hPIV2 V protein targets STAT2 [66]. The specific STAT2 targeting by hPIV2, however, is more promiscuous compared to STAT1 degradation by PIV5 [75]. In fact, infection of BHK cells with hPIV2 led to the specific degradation of STAT1 and not STAT2 [76]. Both STAT1 and STAT2 are required to establish a degradation-permissive environment enabling PIV5 or hPIV2 to target their respective STAT protein. It has been shown that PIV5 and MuV can only target STAT1 in cells that express STAT2 [68,71,77], while hPIV2-mediated STAT2 degradation requires STAT1 [68]. PIV5 V does not bind to STAT1 directly, but it binds directly and independently to both DDB1, a component of a cellular SCF-like ubiquitin E3 ligase complex and STAT2. PIV5 V acts as an adaptor molecule linking DDB1 and STAT1/STAT2 heterodimers, which can ubiquitinate STAT1 in the presence of additional cellular proteins [47,78]. V protein of Mapuera virus (MPRV), a newly emerging *Rubulavirus*, is also an effective inhibitor of IFN signaling, despite relatively low sequence similarities with other *Rubulaviruses.* However, MPRV V proteins do not degrade STATS or affect phosphorylation of STATs, but prevent IFN-induced STAT nuclear accumulation by interacting directly with both STAT1 and STAT2 [79]. Another member of *Rubulavirus*, hPIV4 expresses a V protein that contains a highly conserved cysteine-rich domain. However, hPIV4 V has no effects on STAT protein levels and IFN-induced signaling, even though it can associate with STAT1, STAT2, DDB1, and Cul4A [80]. Therefore, hPIV4 is the only paramyxovirus analyzed to date that can’t evade IFN-induced antiviral responses.

MeV V protein is distinct from V proteins of *Rubulavirus*, sharing only ∼20% overall amino acid sequence identity. However, MeV V protein is known to be an efficient inhibitor of IFN signal transduction in both human and mouse cells, but acts via a distinct mechanism [81]. Like MPRV, the MeV V protein does not degrade STATs, but prevents IFN-induced STAT nuclear accumulation by interacting with components of the IFN-α/β signaling pathway in a multi-protein complex [81,82]. STAT1 and Jak1 have been identified to be direct binding targets of MeV V protein, which results in inhibition of STAT1 phosphorylation by Jak1 [83]. Both N- and C-terminal domains of MeV V protein contribute to the inhibition of IFN-α/β signaling. The binding sites of V to STAT1 and Jak1 are distinct, since STAT1 does not compete with Jak1 for the interaction with V. Residues 110–120 of the V protein have been identified as a minimal STAT1 binding sequence [84]. Mutation at Y110H in the N-terminal domain impairs STAT1 binding, but an additional mutation at C272R in the C-terminal domain is required for abrogation of IFN signaling [83,85–87]. Furthermore, recent studies showed that the C-terminal domain of MeV V interacts directly with STAT2 [84,88]. This interaction with STAT2 is carried by the zinc-finger domain of the C-terminal region. The minimal STAT2 binding peptide contains the cysteine cluster that is a characteristic signature of the V proteins of *Paramyxovirinae*. These three interactions (STAT1, Jak1, and STAT2) allow MeV V to form a multiprotein complex with IFN-α/β signaling components and block signaling downstream of the IFN-α/β receptor. In contrast to the major role of V protein, MeV C protein has only a minor effect on the inhibition of IFN signaling [64,87,89].

In the case of SeV, V protein has no effect on the IFN signaling pathway [90]. Instead, C proteins have been shown to play a major role in blocking IFN signaling [91–95]. SeV expresses a nested set of C proteins, but the shortest C protein, Y2 is fully capable of counteracting the signaling pathway [13]. In fact, anti-IFN signaling activity of C protein does not require the N-terminal 98 amino acids of the protein [96]. A broad range of activities of the C protein has been reported on the mechanism of Jak/STAT inhibition. Some studies showed that C proteins inhibit both STAT1 and STAT2 tyrosine phosphorylation [92,97], and others showed that the C proteins cause prolonged tyrosine phosphorylation of STAT1, and impaired STAT1 serine phosphorylation [98]. It was shown that the dephosphorylation process of pY-STAT1 was also impaired, suggesting that counteraction of IFN signaling by SeV C is caused by disordered phosphorylation and dephosphorylation of STAT1 [98,99]. Actually, SeV C protein physically binds STAT1, and prevents activation of both STAT1 and STAT2 [92,100]. A study using a series of mutant C proteins showed a definite correlation among STAT1-binding capacity and inhibitory activity for IFN signaling [92]. Similar to SeV, the C proteins of other R*espiroviruses* hPIV1 and hPIV3 inhibit activation of STAT proteins [101–103]. Functional mechanisms of anti-IFN signaling of these viruses have not been determined, but a defect in STAT1 phosphorylation in hPIV3 C expressing cells has been reported [103].

## 7. Blocking apoptosis by accessory proteins

When infected with a virus, the host attempts to suppress virus replication in infected cells and viral spread to neighboring cells by various means including induction of apoptosis. Because viruses are intracellular parasites, control over the cell’s death machinery is crucial for viral replication and pathogenicity [104]. Apoptosis is a process in which cells activate intracellular death pathways to terminate themselves in a systematic way in response to a wide variety of stimuli. Apoptosis results from a collapse of cellular infrastructure through regulated internal proteolytic digestion, which leads to cytoskeletal disintegration, metabolic derangement and genomic fragmentation. Many viruses manipulate the apoptotic machinery to their advantage, and in the case of paramyxoviruses, accessory proteins expressed from the P/V/C gene play a role in blocking apoptotic cell death.

Many paramyxoviruses lacking V or C protein expression show enhanced apoptosis in infected cells [21,105–108]. One example is a recombinant PIV5 lacking the C-terminus of the V protein that induces apoptosis in tissue culture, in contrast to wt PIV5 that does not induce cytopathic effect (CPE). This apoptosis induction by the mutant virus can be prevented by expression of wt V, confirming the ability of V protein to prevent PIV5-induced apoptosis [105]. Another study reported that PIV5 containing mutations in the shared N termini of V and P protein (known as CPI–virus) accelerated viral gene expression and caused increased cell death with characteristics of apoptosis, suggesting that multiple regions in V are required for blocking apoptosis [109,110].

In the case of *Respiroviruses,* both hPIV1 and SeV C proteins have been shown to act as inhibitors of apoptosis through the characterization of C-deficient viruses. Early during infection *in vitro*, rhPIV1-P(C–), which is deficient in C protein expression, replicated as efficiently as wt hPIV1, but its titer subsequently decreased coincident with the onset of extensive CPE not observed with wt hPIV1. The rhPIV1-P(C–) infection, but not wt hPIV1 infection, induced caspase 3 activation and nuclear fragmentation in LLC-MK_2_ cells, identifying the hPIV1 C proteins as inhibitors of apoptosis [21]. Similarly, SeV C deletion mutants have been shown to induce apoptosis *in vitro,* and longer C proteins (C’ and C) are more effective for the anti-apoptotic activity [93,107].

Infection with recombinant MeV lacking either V or C also causes more cell death than infection with the parental vaccine-equivalent virus [89,111]. Infection with C-deficient MeV causes significantly less apoptosis in PKR-knockdown cells than in PKR-sufficient cells, suggesting a principal function of the MeV C protein is to antagonize the proapoptotic and antiviral activities of PKR [108].

## 8. Specificity of anti-IFN activities and its role in viral host range

It is clear that anti-IFN and anti-apoptosis activities play a major role in viral pathogenicity to their specific hosts. These activities have also been suggested to contribute to host range restriction because of their specificity. It has been demonstrated that while PIV5 can enter murine cells and initiate viral protein synthesis, the infection is rapidly cleared by the endogenous innate system. PIV5 does not cause STAT1 degradation in murine cell culture [112,113], however, expression of human STAT2 in mouse fibroblasts enabled PIV5 to effectively disrupt murine IFN signaling and to support specific degradation of the endogenous murine STAT1 [114]. These findings demonstrated a unique role for STAT2 as a species-specific host range determinant and corroborated its importance as a cofactor for STAT1 targeting by PIV5. Like PIV5, NDV V protein antagonizes the avian IFN system via STAT1 destabilization [10]. Recombinant NDV deficient in V expression grows poorly in embryonated chicken eggs and chicken embryo fibroblasts (CEFs). Insertion of influenza virus NS1 gene restores impaired growth to wt levels in eggs and CEFs, indicating that NDV V and influenza NS1 are functionally interchangeable. However, in human cells, wt NDV grows poorly compared to NDV V(-)/NS1 virus, suggesting that the anti-IFN activity of NDV V protein is species specific [106]. While the molecular basis has not yet been revealed, it is possible that, like PIV5, avian STAT2 might be involved in this specificity.

Specificity of anti-innate activity was also suggested from studies using closely related *Respiroviruses*, SeV (a murine parainfluenza virus type 1) and hPIV1. A major difference in the anti-IFN activity of these viruses is that SeV expresses both C proteins and a V protein, while hPIV1 expresses only C proteins. A recombinant SeV, rSeVhP, whose P gene was replaced with that of hPIV1, has been rescued. This virus allows the determination of whether hPIV1 C proteins can substitute the function of SeV accessory C and V proteins. rSeVhP infection prevents nuclear translocation of STAT1 upon IFN stimulation, indicating that hPIV1 C protein can inhibit the Jak/STAT pathway in murine cells [102]. Similarly, SeV C protein blocks the IFN signaling pathway in human cells [101], suggesting no specificity in the inhibitory activity of the Jak/STAT pathway between these two viral C proteins. Infection of rSeVhP in murine cells, however, strongly activates IRF-3 and NFκ-B, resulting in an increased level of IFN-β production compared with wt SeV. Insertion of SeV V, but not SeV C gene into rSeVhP partially restores the ability to suppress IFN induction, suggesting that V contributes to the suppression of IFN induction in SeV-infected murine cells [102].

## 9. Concluding remarks

This minireview has illustrated a variety of functions and strategies of paramyxovirus accessory proteins in antagonizing host cell innate activities. Although this group of viruses has similar genome structure and replication patterns, their methods to circumvent the host cell innate activities vary between the viruses of this group. Most of the paramyxoviruses have been shown to antagonize both IFN induction and Jak/STAT signaling pathways by their various accessory proteins. In addition, a study on SeV suggests the accessory proteins can block IFN-independent antiviral activity directly induced in infected cells, the mechanism of which is not known. Much remains to be clarified about the complex interplay between host and viruses, especially, host or tissue specificity and its role in viral pathogenicity and host range. Elucidation of a detailed mechanism is essential for understanding viral pathogenicity and disease development, and will also provide key information that will lead to the development of a safe and effective attenuated live vaccine to clinically important paramyxoviruses.

## Figures and Tables

**Table 1.** Viral interference with IFN production.

Virus	Accessory protein	Cellular target	Mechanism	References
*Respirovirus*				
SeV	V	mda-5	Direct binding to mda-5 blocks activation of mda-5	[32], [34], [52], [53], [61]
	C	RIG-I	Inhibit IRF-3 and NF-kB transduction pathway	[53], [56], [61]
hPIV1	C	ND	Inhibit IFN production stimulated by hPIV1 infection	[62]
bPIV3	V	mda-5	Direct binding to mda-5 blocks activation of mda-5	[53]
	C	ND	Suppress dsRNA-stimulated IFN production	[53]
*Morbillivirus*				
MeV	V	mda-5	Direct binding to mda-5 blocks activation of mda-5	[34], [52]
*Rubulavirus*				
PIV5	V	mda-5	Direct binding to mda-5 blocks activation of mda-5	[32], [34], [52]
hPIV2	V	mda-5	Direct binding to mda-5 blocks activation of mda-5	[32], [52]
MuV	V	mda-5	Direct binding to mda-5 blocks activation of mda-5	[32], [52]
*Avulavirus*				
NDV	V	mda-5	Direct binding to mda-5 blocks activation of mda-5	[52]

ND, not determined.

**Table 2.** Viral interference with IFN signaling.

Virus	Accessory protein	Cellular target	Mechanism	References
*Respirovirus*				
SeV	C	STAT1	Disordered phosphorylation of STAT	[13], [90–99]
hPIV1	C	ND	Prevent nuclear translocation of STAT	[100], [101]
hPIV3	C	ND	Block phosphorylation of STAT	[102]
*Morbillivirus*				
MeV	V	STAT1, Jak1	Block phosphorylation of STAT1	[80–87]
		STAT2		
*Rubulavirus*				
PIV5	V	STAT1	Proteasome mediated degradation of STAT1	[47], [65], [68], [70], [75], [77], [78]
hPIV2	V	STAT2	Proteasome mediated degradation of STAT2	[66], [68], [70], [75], [76]
MuV	V	STAT1, STAT3	Proteasome mediated degradation of STAT	[67], [69], [71], [72], [74]
MPRV	V	STAT1, STAT2	Prevent nuclear translocation of STAT	[79]
*Avulavirus*				
NDV	V	STAT1	Proteasome mediated degradation of STAT1	[9]

ND, not determined.
